# Supplementary material for: System Analysis Based on Lipid-Metabolism-Related Genes Identifies AGT as a Novel Therapy Target for Gastric Cancer with Neoadjuvant Chemotherapy
Source: Pharmaceutics. 2023 Mar 2;15(3):810. doi: 10.3390/pharmaceutics15030810 (PMC10051152; doi:10.3390/pharmaceutics15030810)
Supplement: Supplementary file 1 [file pharmaceutics-15-00810-s001.zip › Supplementary Figure.pdf]

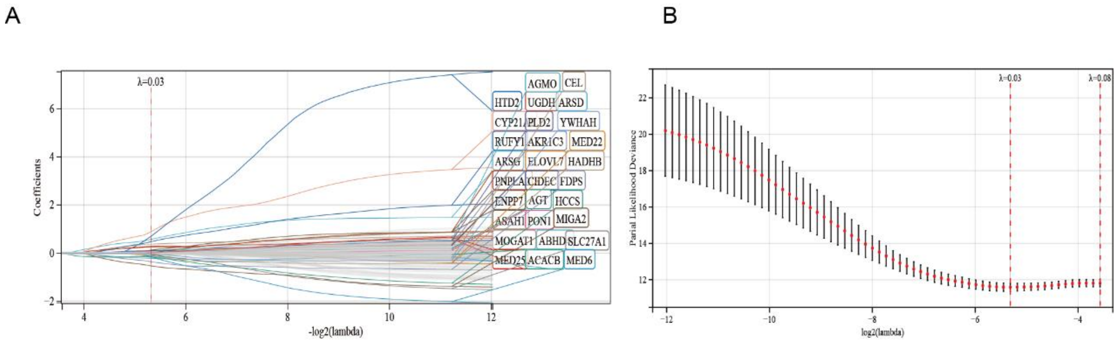

Supplementary Figure S1. Construction of risk model in the TCGA cohort. (**A**,**B**) LASSO coefficient profile analysis and cross-validation to identify the most useful prognostic genes.

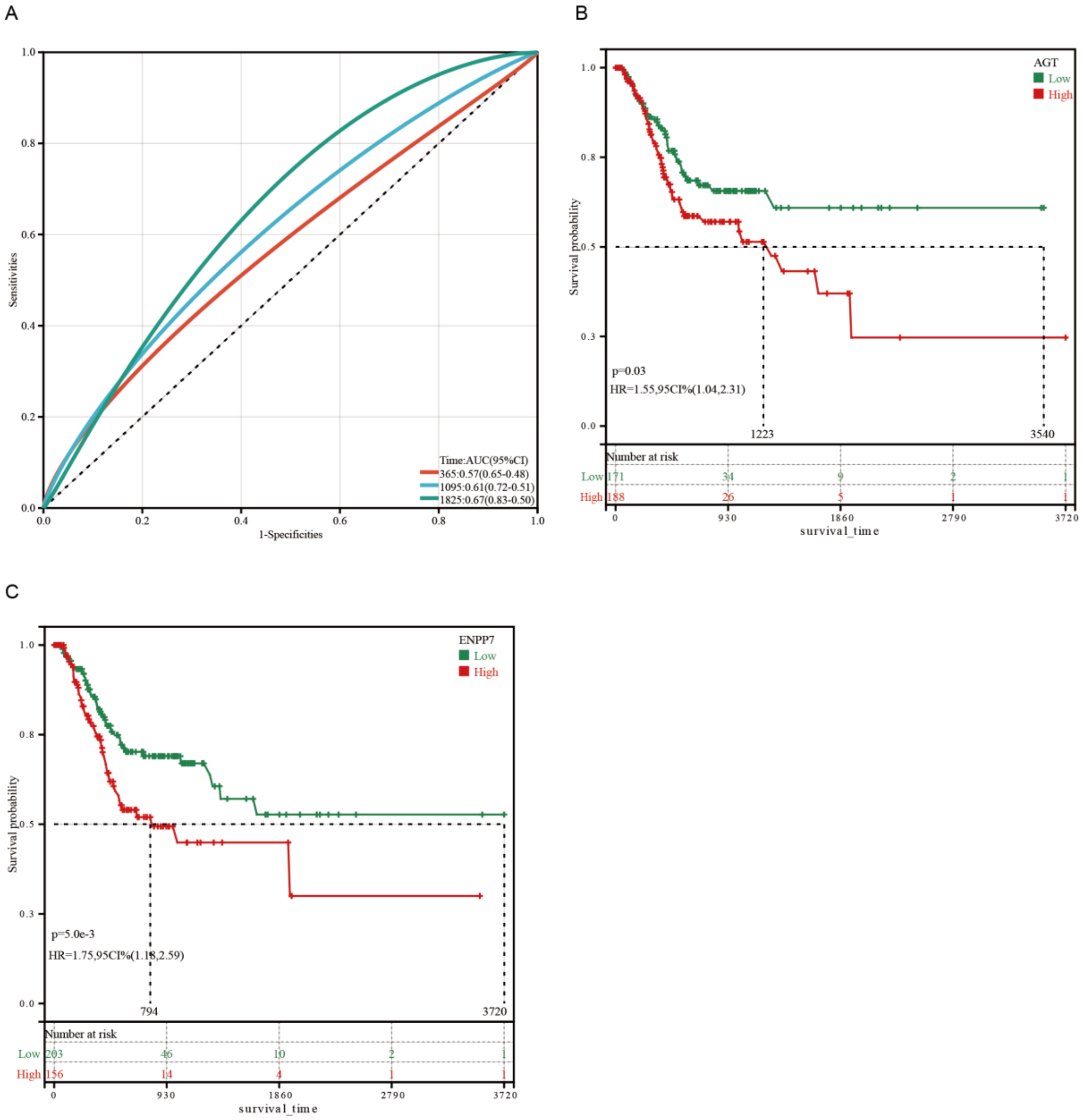

Supplementary Figure S2. ROC curve plot and survival plot. (**A**) ROC curve of the risk model in the TCGA cohort at 1, 3, and 5 years. (**B**,**C**) Kaplan–Meier curves of OS in the TCGA cohort based on the expression of AGT and ENPP7, respectively.

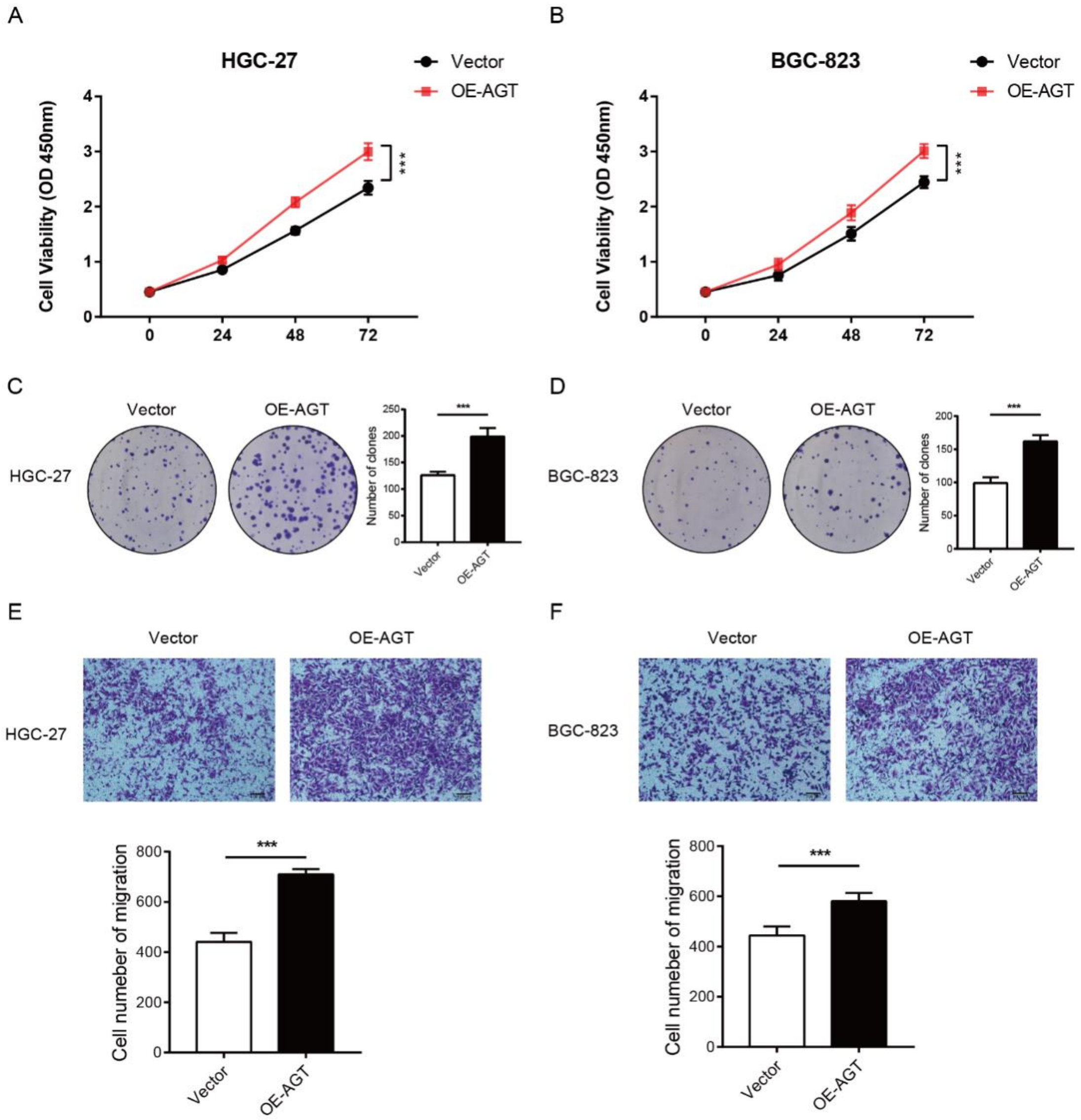

Supplementary Figure S3. AGT promoted the cell proliferation and cell migration in GC cells. (**A**,**B**) Cell viability was determined by CCK8 assay in the HGC-27 and BGC-823 cell lines transfected with vector or OE-AGT. (**C**,**D**) Colony formation assay of HGC-27 and BGC-823 cell lines transfected with vector or OE-AGT. (**E**,**F**) HGC-27 and BGC-823 cells were grown and transiently transfected with vector or OE-AGT and then subjected to Transwell for 24 h. *** $p < 0.001$.

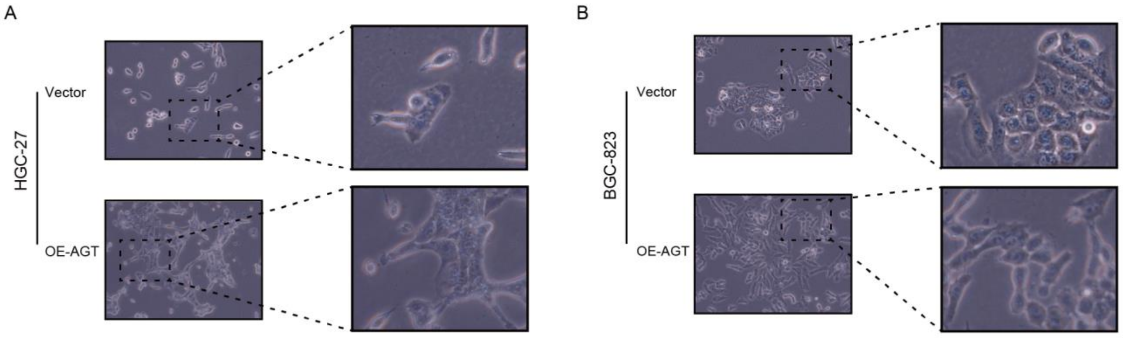

Supplementary Figure S4. The AGT promoted the EMT phenotype transition in HGC-27 and BGC-823 cells.
(**A**,**B**) The morphology of HGC-27 and BGC-823 cells transduced with Vector or AGT are shown.

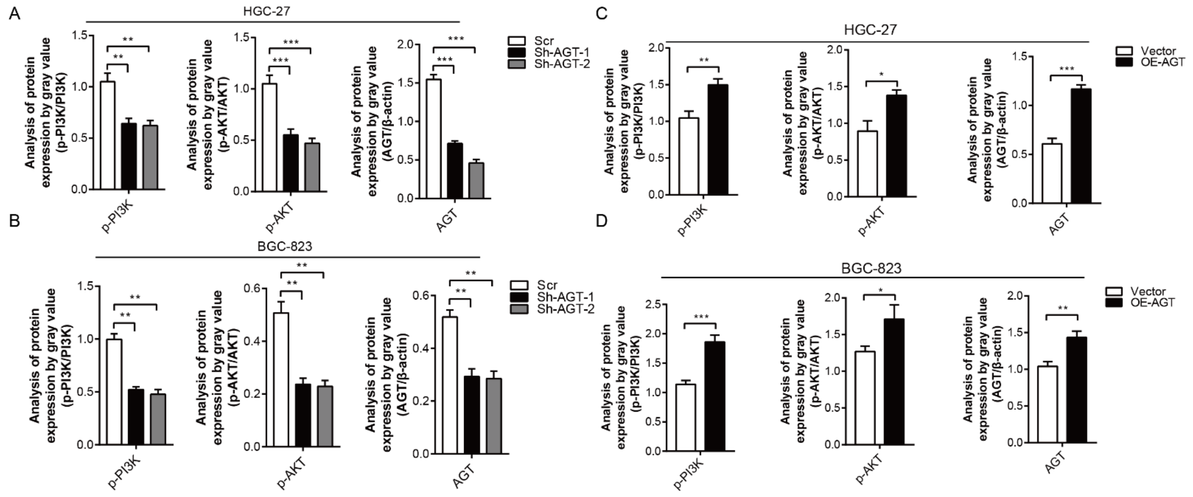

Supplementary Figure S5. Relative expression levels of p-PI3K, p-AKT, AGT in the HGC-27 and BGC-823 cells in indicated conditions.
(**A**–**D**) All the statistical figures of gray values corresponding to Figure 7E,F, respectively. *** $p < 0.001$, ** $p < 0.01$, * $p < 0.05$.

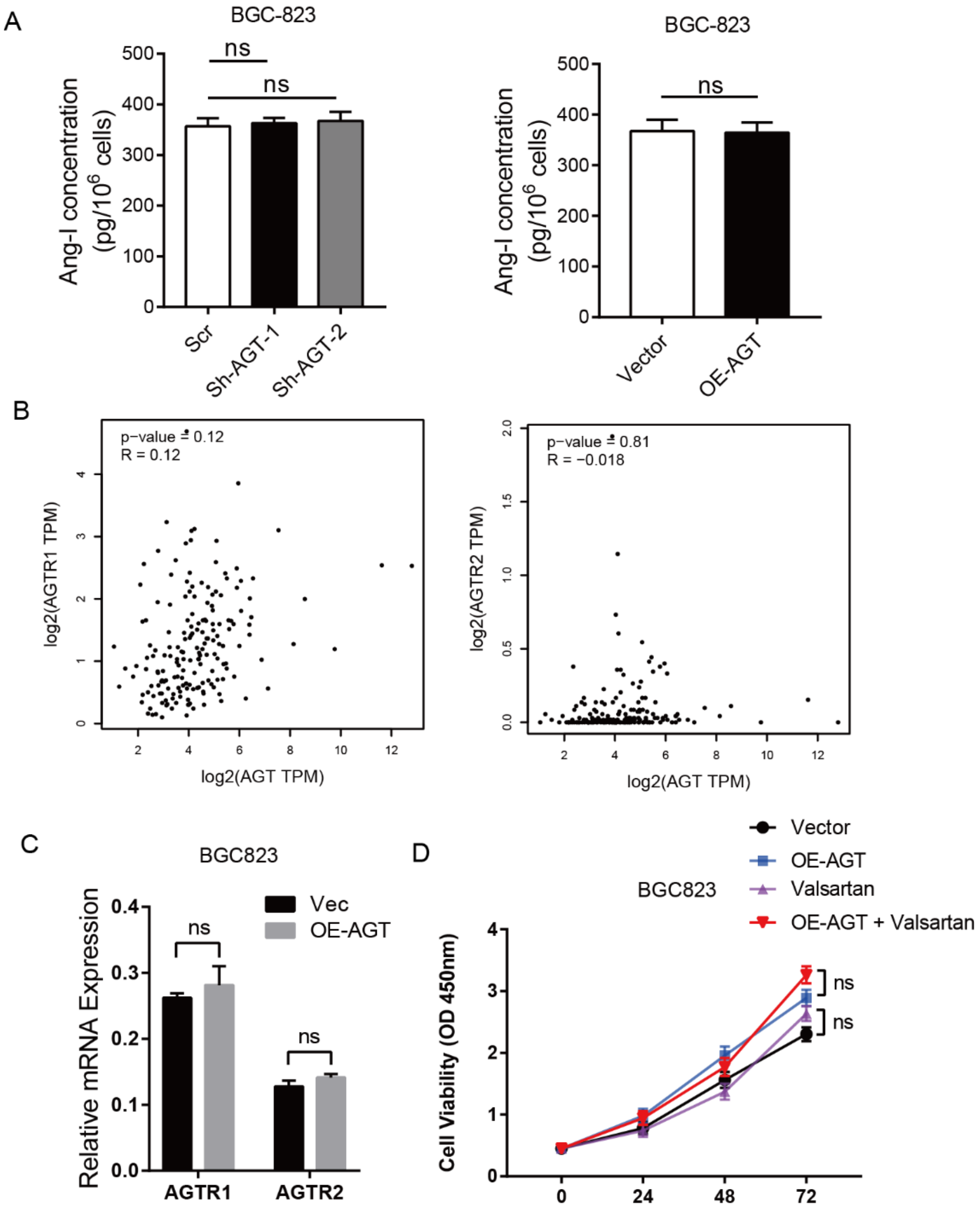

Supplementary Figure S6. The relationship between AGT and renin-angiotensin system in GC cells. (**A**) ELISA assays for Ang-I in the supernatant of BGC-823 cell line which treated in the indicated conditions. (**B**) The correlation between AGT and AGTRs, was analyzed in TCGA dataset using GEPIA web tool. (**C**) The relative mRNA expression level of AGTR1 and AGTR2 in BGC-823 cell line that transduced with OE-AGT plasmid. (**D**) Cell viability was determined by CCK8 assay in the BGC-823 cell line which treated with Valsartan (10^{-6} mol/L) or OE-AGT plasmid. ns, no significance.

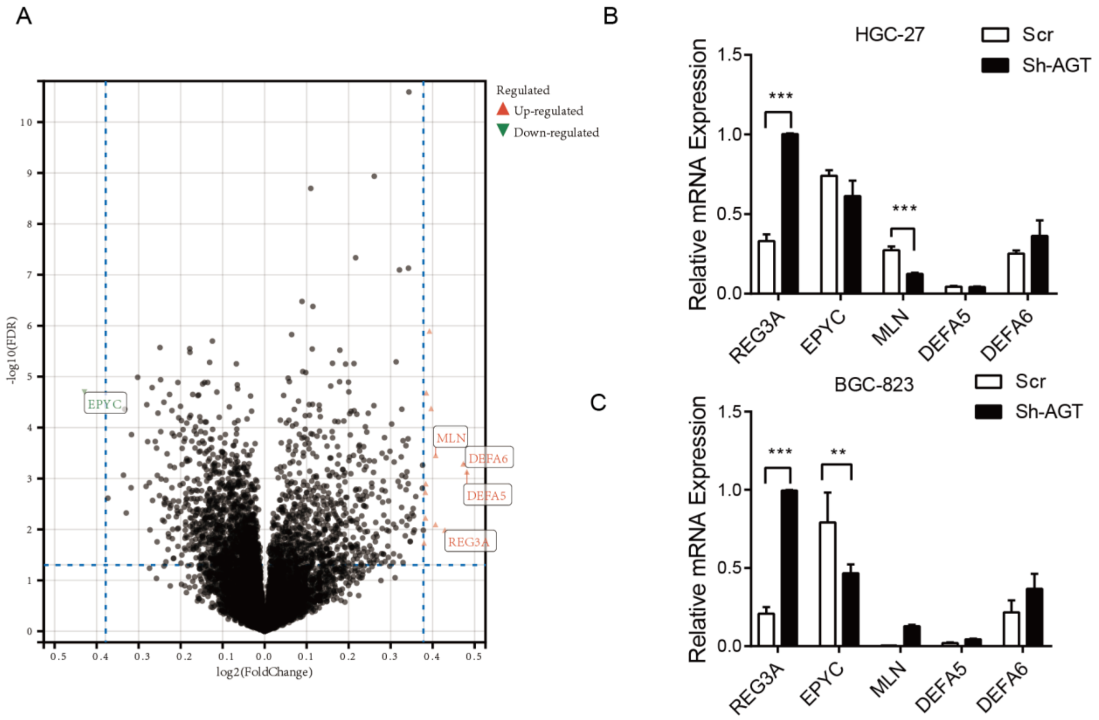

Supplementary Figure S7. The DEGs between high/low-AGT expression groups and relative mRNAs expression in GC cells.
(**A**) Differentially expressed genes in high AGT expression level and low AGT expression level groups. Red and green dots indicate upregulated and downregulated genes, respectively. (**B**,**C**) The relative mRNA expression level of REG3A, EPYC, MLN, DEFA5, and DEFA6 in HGC-27 and BGC-823 cells which transduced with Scr or Sh-AGT. *** $p < 0.001$, ** $p < 0.01$.
